# The impact of congenital cytomegalovirus infection among families and caregivers: A qualitative analysis of responses to a public consultation on newborn screening in the UK

**DOI:** 10.1177/13558196251382548

**Published:** 2025-10-09

**Authors:** Rosamund Greiner, Sarah Dewar, Christine E. Jones, Marthe Le Prevost, Tushna Vandrevala, Cristina Visintin, Heather Bailey

**Affiliations:** 1 4919 Institute for Global Health, University College London (UCL), London, UK; 2 CMV Action, UK; 3 Faculty of Medicine and Institute for Life Sciences, 7423 University of Southampton and NIHR Southampton Clinical Research Facility and NIHR Southampton Biomedical Research Centre, University Hospital Southampton NHS Foundation Trust, Southampton, UK; 4 MRC Clinical Trials Unit, University College London (UCL), London, UK; 5 Centre for Applied Health and Social Care Research, Faculty of Health, Science, Social Care and Education, 7423 Kingston University, Kingston-Upon-Thames, UK; 6 UK NSC Secretariat, London, UK

**Keywords:** screening, cytomegalovirus, policy, family carers

## Abstract

**Objectives:**

To describe the impact associated with congenital cytomegalovirus (cCMV) infection and experiences and perceptions of people with experience of CMV in pregnancy and families / caregivers of children diagnosed with cCMV, who responded to a UK National Screening Committee (UK NSC) public consultation on cCMV screening.

**Methods:**

The public consultation was conducted in 2021-22 on a draft evidence review and was aimed at informing the UK NSC’s decision on newborn screening for cCMV. Data were analysed using framework analysis: a subgroup of responses was inductively coded, codes were refined and initial themes identified, before targeted coding of the remainder of the data and identification of final themes and sub-themes.

**Results:**

Of a total 155 responses, 125 (describing 128 pregnancy/child outcomes) contained information relevant to the coding framework and were included. Most (n = 109) described a live birth of a surviving child, of whom 90% (98/109) were living with symptoms or long-term sequelae of cCMV at the time of the response. Two main themes were identified: missed opportunities and emotional impacts attributed by respondents to not screening for cCMV. Many families described delays in their child’s cCMV diagnosis, including due to healthcare professionals’ lack of awareness of cCMV, and viewed newborn screening as a solution to avoid delays in diagnostic pathways. Diagnostic delays resulted in a lasting sense of injustice and unfairness due to possible missed opportunities to improve outcomes (e.g., through antiviral treatment or early therapies), as well as uncertainty and anxiety.

**Conclusions:**

Responses were predominantly from parents and caregivers of children with cCMV who experienced long term disability. They highlight significant gaps in awareness, support and health care for affected children that need addressing, regardless of national screening policy decisions. These responses contribute to the literature on lived experiences of individuals and families affected by cCMV.

## Introduction

In high-income countries, an estimated 0.5% of babies are born with congenital cytomegalovirus (cCMV),^1^ of whom 17%–20% have long term sequelae which can include hearing loss, neurological and developmental impairments.^2^ Long term sequelae are more common in those infants with symptoms or signs at birth, affecting around half of these infants, compared to 10%–15% of infants with no clinically detectable features of cCMV at birth.^2^ Applying these percentages to 560,000 annual births in England equates to an estimated 2800 infants born with cCMV each year (using the 0.5% average birth prevalence found in high income countries; current birth prevalence specific to England is uncertain). Of these, 560 will have long term sequelae from the infection (210 born with and 350 born without cCMV-related symptoms). Congenital CMV infection is the cause of 10%–20% of permanent childhood hearing loss overall.^3,4^ At its most severe, cCMV disease can cause the devastating impacts of pregnancy loss, stillbirth and child death, with 0.5% of infants born with cCMV estimated to die due to the infection.^2^

CMV infection is not routinely screened for during pregnancy or among newborns in the UK, so diagnosis of cCMV typically follows suspected or confirmed maternal infection, identification of ultrasound findings indicative of fetal infection, or symptoms or signs in the infant or child. Antiviral treatment is indicated for infants less than 1 month of age who have significant symptoms and signs of cCMV.^5^ This is based on findings from two randomised controlled trials, which found modest benefits of antiviral treatment for preservation of hearing and for neurodevelopmental scores.^6,7^ Treatment with antivirals is also recommended in European guidelines for children with isolated sensorineural hearing loss (SNHL), based on a non-randomised trial that showed potential benefit when initiated up to 12 weeks of age.^5,8^ However, in a randomised controlled trial, later initiation of treatment over a wider age range (1 month to <4 years) did not improve hearing outcomes among children with cCMV-associated SNHL, most of whom had symptomatic cCMV disease.^9^ Prompt diagnosis of cCMV in these children is therefore of crucial importance to inform decisions around treatment, follow-up, rehabilitation and support. This has led to the introduction of early cCMV detection pathways (e.g. targeted CMV saliva screening of infants referred to audiology following newborn hearing screening) in some areas of England.^10,11^

The UK National Screening Committee (UK NSC) does not currently recommend universal newborn screening for cCMV (based on a review carried out in 2017 and updated in 2021) due to: evidence gaps around appropriate screening tests; lack of reliable prognostic factors for long term outcomes; uncertain consequences of identifying cCMV infections in children who are likely to have minimal or no symptoms (around 4 in 5 children with cCMV overall); and an overall lack of evidence that universal newborn screening for cCMV would improve outcomes.^12,13^ In recent years, universal newborn screening has been implemented in several provinces and states in North America.^14^

Screening during pregnancy was also not recommended in the most recent UK NSC evidence review, due to lack of a reliable screening test and lack of an intervention to prevent vertical transmission or minimise cCMV infection severity.^12^ Since that review, an evolving evidence base has shown that valaciclovir given during pregnancy can reduce the risk of vertical transmission of CMV among women with a primary infection in the periconception period and first trimester.^15^ The possibility of shifting approach from treatment to prevention of cCMV has led to an increased interest in antenatal screening in some high-income countries, and a 2024 recommendation from the European Congenital Cytomegalovirus Initiative for universal CMV serology screening among women not known to be seropositive to be considered.^5^ However, most guidelines do not currently include recommendations for antenatal CMV screening, and research gaps remain around its population level risk/benefit and impact on cCMV-related sequelae in infants and children.^16^

A small number of qualitative, survey and health economic studies have explored the perspectives and experiences of children affected by cCMV and their families. They show poorer health related quality of life correlated with severity of long-term cCMV-related impairments among children and their parents, and highlight the wide-ranging impacts of caregiving on physical and mental health, work opportunities, finances and relationships of parents and family.^17–21^ These studies also emphasise the specific challenges posed by delays in diagnosis and lack of awareness of cCMV among healthcare professionals and support networks. These experiences are likely to be shaped by factors including heterogeneous impacts of cCMV, context of health systems and policy (including screening policy), and cultural attitudes to disability, including the ways in which ableist attitudes and language may impact on social relationships and support of parents and children affected by cCMV.^19^ A recent paper published by three authors who are public health and healthcare professionals and also mothers of children with cCMV, state: “*It is important to note that while we do not view our children as a burden, we also feel that it is important to acknowledge the reality of cCMV and its potential impacts on families”* ^19^. They highlight the need for the lived experiences and indirect effects of cCMV on families and caregivers to be better understood and incorporated into health policy decision making.^19^

The aim of this work is to explore the perceptions and experiences of parents, families and others with personal experience of CMV in pregnancy or a child diagnosed with cCMV, through analysis of written responses to a UK NSC public consultation on cCMV screening policy conducted in 2021. These responses were submitted as part of the UK NSC’s decision-making process and were taken into account in the decision not to introduce universal newborn screening for cCMV.

## Materials and methods

### UK NSC consultation

The UK NSC assess evidence for a broad range of conditions against a set of criteria to provide advice to ministers and the NHS about all aspects of targeted and population screening.^22^ Evidence is re-evaluated periodically to determine whether any changes should be made to existing screening recommendations. The most recent full reviews for antenatal and newborn CMV screening were conducted in 2012 and 2017 respectively. In 2021, the UK NSC commissioned an evidence map on newborn screening only. The production of such rapid evidence products is an important step in the UK NSC’s policy making process. The evidence map concluded that the volume and type of new evidence published since the 2017 review was insufficient to justify a new full review, or to change the conclusion that newborn screening for CMV does not meet the criteria for population screening.

The evidence map findings were made available for public consultation over a 3-month period (28 October 2021 to 17 January 2022), hosted on the UK NSC website. Stakeholder groups and individuals were invited to respond; members of the public were asked to respond to the following six questions.

1 Please tell us if this condition has affected you, your family or your friends?
2 Do you have any comments on the evidence considered by the UK NSC in the review? For instance, was any important evidence missed?
3 Do you have any comments on the discussion, conclusion or recommendation in the review?
4 Do you think screening should or should not be recommended? Why?
5 There could be many alternatives to a screening programme. How else do you think the NHS or the government could help people with the condition?
6 Do you have any other recommendations?

Demographic information about the respondents was not collected. None of the questions required mandatory responses. 155 members of the public with personal experience of CMV responded to the consultation, and their responses were anonymised and made publicly available on the NSC website.

A final version of the evidence map incorporating post-consultation revisions was published in June 2022.^13^ In this paper, we considered for coding all consultation responses from individuals who responded in a personal capacity, the large majority of whom were parents of children affected by cCMV.

### Data management and analysis

In order to prepare the data set for coding, the anonymised publicly available data were split into 155 separate responses, and the respondents were numbered (1-155). Data familiarisation also occurred at this phase, as the researcher read through the responses in the process of preparing the data set for analysis. The data were then imported into Dedoose for line-by-line coding (Version 9.2.014, www.dedoose.com). The circumstances through which the respondent had been affected by cCMV were extracted from each response, where available (for example, whether they were a parent of a child or had experienced a pregnancy loss). For respondents referring to a child affected by cCMV, their relationship to the child was also recorded for each response in a spreadsheet. The purpose of recording this data was to develop a profile of the respondents.

The data were analysed using a framework analysis which involved the inductive development of a coding framework based on a subset of the data, followed by the systematic application of this framework to the whole dataset.^23^ Framework analysis provides a structured approach to qualitative data analysis which is particularly useful for collaborative research in interdisciplinary teams.^23^ The first stage of data analysis was inductive coding. A sample (10%) of the total responses were purposively selected for coding at this stage. Length of response was used as a proxy for richness, and the 12 longest responses were selected for this phase. These 12 responses were supplemented with three responses that discussed less common outcomes (termination, pregnancy loss, infant death) to capture the diversity of responses in the initial coding. In total, 15 responses were inductively coded.

The second stage of the analysis was refinement of codes and identification of themes based on the first round of inductive coding. This stage also involved investigator triangulation as RG presented initial codes and selected representative quotes to CJ, HB, TV and MLP. Through discussion, a more refined coding framework was developed; some related codes were merged, and codes that captured too broad a range of ideas and sentiments were split into smaller, more specific codes. CJ, HB, TV and MLP also drew on their subject matter expertise on CMV and infections to refine the coding framework, e.g., by illuminating subtle but important differences between related codes and divergences between respondent beliefs and the current clinical evidence. Broad initial themes in the data were also identified at this stage.

The third stage of the analysis was targeted coding of the remaining responses, using the refined coding framework. Responses that contained no data related to the established coding framework were noted. Code labels were iteratively developed throughout the coding process to best represent the content of each code.

Finally, once all of the responses had been coded, codes were organised and mapped to identify relationships between them and refine the broad themes that were identified in the second stage of analysis, before final over-arching themes and sub-themes were agreed. Quotes that were selected for inclusion in the article were edited for spelling and grammar to ensure their meaning was clear.

## Results

### Respondent profile

Of the 155 responses to the consultation from people responding in a personal capacity, 125 contained information relevant to the coding framework, and these responses were coded. The remaining 30 responses contained no data relevant to the coding framework and were excluded. The 125 responses that were coded referred to 128 outcomes, as two respondents discussed the birth of twins, and one discussed two separate pregnancies.

Of the 128 outcomes described in the coded responses, most referred to the live birth of a surviving child (n = 109, 85%). Of these, 90% were living with symptoms or long-term sequelae of cCMV at the time of the response (n = 98), while only 5% (n = 5) remained asymptomatic at the time of the response. For the remaining 6% of live births, it was unclear from the response whether the child was living with symptoms or long-term sequelae of cCMV (n = 6). Respondents referenced a wide range of neurodevelopmental symptoms including sensory, cognitive and motor impairments and neurodiversity, as well as those whose children had no impairments.

There were a small number of responses that discussed pregnancy loss (n = 5, 4%), termination of pregnancy (n = 5, 4%), infant death (n = 4, 3%), and current pregnancies (n = 3, 2%). In the remaining 2% of responses, the outcome of the pregnancy discussed by the respondent was unclear (n = 2).

The age of the child at the time they were diagnosed with cCMV varied widely among the responses. A total of 8% of responses discussed receiving a diagnosis during pregnancy (n = 10), while in 15% of responses the diagnosis was made within the first 4 weeks of life (n = 19). A further 5% were diagnosed between 4 weeks and 3 months of age (n = 6), and 16% were diagnosed after 3 months of age (n = 21). In the remaining 40% of responses, the child’s age at the time of cCMV diagnosis was unclear based on the response (n = 51).

The majority (74%) of respondents specified that they were parents or primary caregivers to a child living with cCMV (n = 93). 16% of respondents specified that they were grandparents to a child living with cCMV (n = 20), and 5% specified that they were an aunt or uncle (n = 6). The remaining respondents were friends of the family, other relatives, or did not specify their relationship (n = 6, 5%).

### Findings

**Figure 1.**
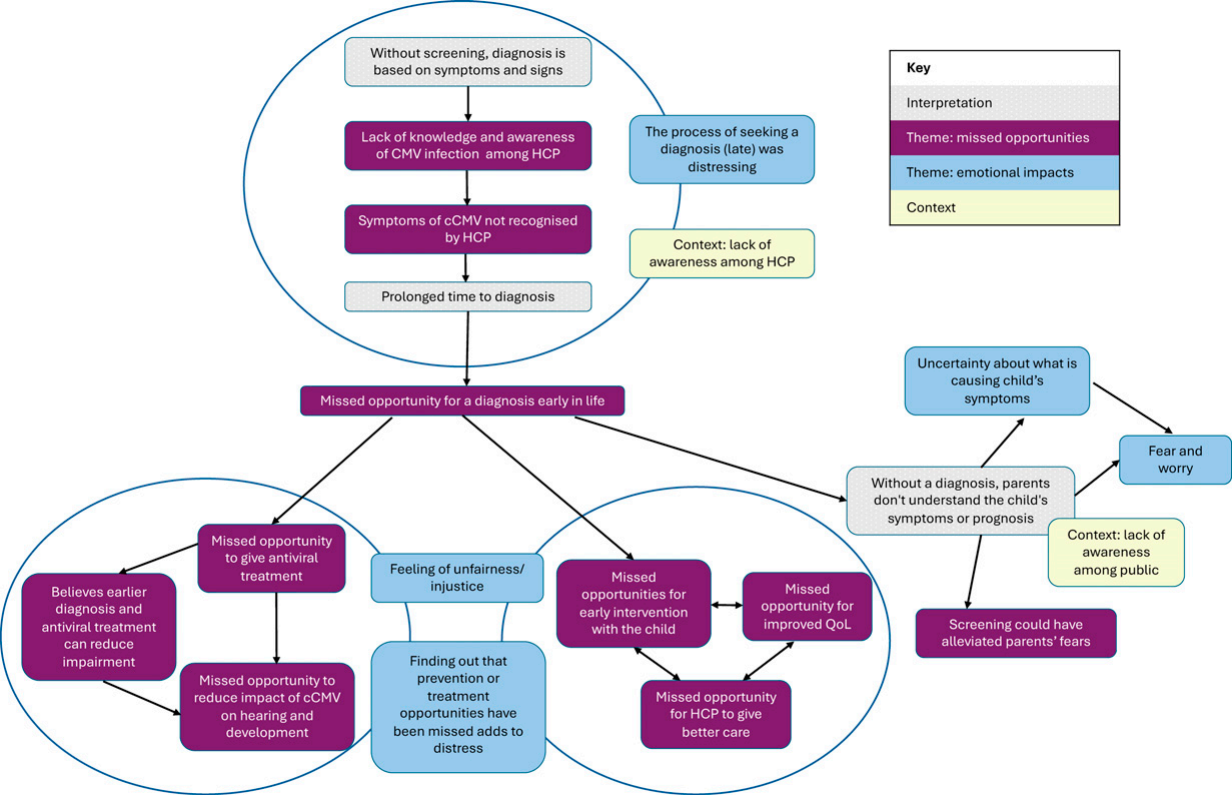
Diagram showing the relationship between themes and sub themes identified through analysis.

Two interconnected overarching themes were identified: missed opportunities and emotional impacts attributed by respondents to not screening for cCMV. Respondents felt that not screening for cCMV led to a series of missed opportunities to improve outcomes for their child and their family. These missed opportunities were associated with a range of challenging emotions. The relationships between these themes are shown in Figure 1.

### Missed opportunities for a timely diagnosis of cCMV

#### Seeking a diagnosis was a slow process

Many respondents pointed out that in the absence of screening, diagnostic testing for cCMV is prompted by clinical features. Owing to a lack of awareness and knowledge of cCMV among healthcare professionals they encountered, symptoms and signs were sometimes missed which delayed the diagnosis of cCMV. The experience of Respondent 86, a parent of a child living with cCMV, was characteristic of many respondents’ experiences of their child’s first days and weeks of life. They said:

“My daughter was not diagnosed until she was 5 weeks old even though she was very poorly when she was born and spent 2.5 weeks in NICU. Doctors never mentioned the possibility of cCMV and seemed confused as to the reason why she was so poorly. She had all the classic symptoms of a baby born with cCMV but as this is not screened for, it was not picked up until she failed her hearing test.”

In the context of limited awareness of cCMV among healthcare professionals, respondents believed that screening would result in the diagnosis of cCMV being made more quickly. Respondent 17, a parent of a child diagnosed with cCMV after 10 weeks of age, stated:

“Not enough medical staff are aware of the symptoms to begin assessments after birth when necessary. If the screening was universal, it would be picked up more efficiently.”

Respondents also discussed a feeling of not being taken seriously in hospital despite it being apparent to them that their child was not well. Respondent 57, a parent to a child diagnosed with cCMV at 7 months of age, stated that

“From the word go my pregnancy was a very difficult one. I was up at our local hospital constantly with worry and concerns, I felt totally disregarded. [Daughter] was born with many of the signs and symptoms of CMV, she was overlooked.”

Respondents viewed each of these barriers as contributing factors to delay in confirming a diagnosis.

#### The process of seeking a diagnosis for their child was distressing

Respondents found the (sometimes lengthy) process of seeking a diagnosis for their child’s symptoms distressing. Referring to the process of getting a diagnosis for their son, Respondent 128 said:

“It has been a very long, drawn out and traumatic process for us all.”

In the absence of a confirmed diagnosis, some respondents pondered different possible scenarios and experienced anxiety and fear about what the diagnosis might be. Respondent 71’s child was not diagnosed with cCMV until they were 4 years old. They stated that:

“The years which followed [my son’s birth] and awaiting the diagnosis were filled with many medical appointments... It was a bewildering and frightening time as differing potential diagnoses were considered including possibly degenerative conditions.”

As this quote illustrates, part of the respondents’ distress also stemmed from having to attend numerous medical appointments and the anxiety related to an unknown condition with potentially serious implications for their child’s health. Respondents also reported that the process was intrusive to the children, which added to the caregiver’s discomfort. Respondent 100, a custodial grandparent of a child diagnosed with cCMV at 20 weeks of age, reflected on the impact of the diagnosis process on their child.

“I noticed she can’t hear, after investigation she is deaf in one side and partial hearing in other. She was tested for CMV at 20 weeks, and it was found. She is going though lots of other tests as well, this could have all been sorted a lot quicker if she was tested when she was born, and she wouldn’t have to be still going through so much now.”

In the context of a distressing and often slow process of diagnosing cCMV, respondents believed that not screening for CMV results in a missed opportunity for a timely diagnosis, in turn resulting in further missed opportunities for intervention and support and emotional consequences for those living with the impact of cCMV-related sequelae on their child.

#### Screening could have alleviated parental fears and anxiety

Without a diagnosis, respondents were unable to understand the prognosis for the child or seek treatment for their symptoms. This created lasting uncertainty and fear about how the child’s condition might develop or deteriorate with time. Respondent 29, a parent of a child with symptoms of cCMV identified in utero described their emotional distress while they awaited a diagnosis.

“Routine screening for this virus can prepare a parent in the same way every other screen does... I.. was told at my 20wk scan my baby had almost 0 chance of survival. All other tests were done and came back negative therefore I was left with no answers to what was wrong through the rest of my pregnancy. What was already the worst and scariest time of my life was made worse by not knowing why.”

Respondents saw screening as a way in which these fears could have been alleviated, by providing an earlier diagnosis which would explain the child’s symptoms, and providing clarity about how the condition should be managed. Respondent 14, a parent of a child living with cCMV, put it like this:

“Screening in pregnancy would have lessened a lot of the worry, stress and turmoil my family have gone through.”

### Without a timely diagnosis, treatment opportunities are missed

#### Missed opportunity to administer antiviral treatment

Once family members learned about cCMV and its effects on child development, they also became aware of possible treatment options. Many respondents discovered the diagnosis only after the first month of life, missing the critical window during which initiation of antiviral treatment has been shown to be effective in trials. As respondent 47, a grandparent to a child living with cCMV, put it*:*

“My granddaughter was diagnosed at around 18 months with CMV, we only knew because they went back and tested her heel prick from birth, by that point it was too late for her to have antivirals.”

Respondents believed that if their child had been screened for CMV, antiviral treatment could have been initiated immediately. Respondent 38’s child was diagnosed with cCMV at the age of 4 years old. They echoed many of the responses about the missed opportunity for antiviral treatment due to the lack of screening for cCMV, saying,

Screening should be done. If we would have known at birth about her CMV then we could have started antivirals or medication to stop the impact of CMV!”

#### Missed opportunity to avert more serious impairment

Respondents believed that screening for cCMV at birth would facilitate the early initiation of treatment, which might have reduced the long-term impact of cCMV on their child. Respondent 18 said,

“When I was pregnant with my daughter, I caught CMV and wasn’t aware I had it. She was born deaf in her right ear and when she was 6 her hearing in her left ear which was perfect at birth started to deteriorate... If she had been tested at birth this later onset deafness could have been avoided.”

Respondents thus viewed the lack of screening and slow process of getting a diagnosis of cCMV as a missed opportunity to avert more serious impairment in children with cCMV. Respondent 65, a grandparent to a child living with cCMV had a similar point of view:

“Screening should be recommended. The delay in a baby’s diagnosis prevents antivirals being administered in good time and these may reduce some of the bad effects.”

#### Learning of missed opportunities contributes to distress

Learning that prevention or treatment opportunities have been missed added to the distress experienced by families. Respondent 78, a parent of a child living with cCMV, said,

It was heartbreaking to be told that he could have had some help if we had known, yet we only found out far too late.”

Respondents expressed a shared belief that their lives and their children’s lives could have been different if treatment was given within the first 4 weeks of life. Respondent 64, a parent, said,

“Even though only minority of children born with cCMV end up having such a hard life, it surely doesn’t feel very minor to the families. It is traumatic and painful to have such a different life to what we were hoping for. Especially if it could have been helped.”

### Missed opportunities for improved quality of life

#### Missed opportunities for better care and early intervention

The long process of seeking a diagnosis also meant that respondents felt that opportunities for early intervention with their child were missed. Many respondents believed that if children had a diagnosis at birth, this would promote access to specialist medical care and monitoring, therapeutic interventions, and other support to promote development. Respondent 79, a parent of a child living with cCMV said of their daughter,

“Although she had symptoms, she still wasn’t tested when she should have been. If routine testing was in place, [daughter] could’ve had the option to receive antivirals and early intervention therapies such as speech and language, physiotherapy etc. But this didn’t happen for her.”

Respondent 109, an aunt or uncle to a child with cCMV, concurred, saying

“This condition has affected my niece who has hearing loss as a result. She is one of the lucky few that has mild symptoms however this was not picked up until she was around 5 years old! If known sooner, she could have been supported from a much younger age and interventions could have been implemented much earlier.”

#### Missed opportunity for improved quality of life for families

Based on the missed opportunities for treatment and early intervention, respondents identified that opportunities for a better quality of life for children with cCMV and their family members had been missed. Respondent 46, a parent, said,

“Had there been greater awareness of CMV and its effects, and in particular had CMV been screened for at the appropriate time, then our lives as a family might be very, very different and dramatically improved.”

Uncertainty around the cause of the child’s symptoms, the long process of seeking a diagnosis, and the missed opportunity for possible treatment all had a detrimental effect on family quality of life. This is apparent in the response of another parent, Respondent 51, who said,

“Saliva and urine testing should also be considered... to ensure earlier diagnosis and management. This would have without doubt improved our son’s quality of life and ours earlier and improved his outcomes.”

It is evident in the responses that quality of life for children, caregivers, and other family members was highly interrelated.

### Feelings of injustice at lack of screening

The series of missed opportunities that respondents identified as stemming from the policy of not screening for CMV culminated in feelings of injustice. Discussing the policy of not screening for CMV, Respondent 100, a custodial grandparent, said,

“It’s not fair on the children or the families.”

Respondents pointed to other, less common conditions that are routinely screened for, and questioned why the same policy would not be extended to include cCMV. Respondent 113, a parent, said,

“Screen at birth! More growth scans during pregnancy to monitor growth and development! More public knowledge about the condition, there’s adverts for heart disease and diabetes, what about cmv!!! My child needed help, the NHS failed!”

As is evident in this quote, respondents felt let down by the lack of awareness of cCMV among the public and healthcare professionals, and by the lack of screening. They felt that the missed opportunities were unfair on them and their child. Respondent 112, a woman who experienced pregnancy loss at 31 weeks’ gestation due to cCMV, expressed her disappointment and grief:

“Had they been screened, they could have monitored him and intervened before his prognosis was death. Surely these babies’ lives should matter just as much as anyone else’s? I’ve dedicated my career to the NHS helping others, but where was my help in a simple blood test? How would you feel if this was you or your family. It can affect anyone, please stop this senseless heart ache and screen for CMV.”

## Discussion

This paper presents a novel analysis of public responses to a UKNSC consultation, a key step in the UKNSC’s policy making process. Our analysis demonstrates the value of secondary analysis of qualitative data in illuminating families’ lived experiences, with implications for policy and health services research, as has been suggested elsewhere.^24^ Our analysis of 125 consultation responses, predominantly from parents or caregivers of children living with disability caused by cCMV, highlights how respondents attributed a wide range of missed opportunities and emotional impacts to the lack of screening for CMV (newborn screening and, to a lesser extent, antenatal screening). Although the majority of children with cCMV do not have sequelae, almost all respondents to this consultation had personal experience of a child with long term consequences of cCMV or adverse outcomes related to cCMV, including infant death, pregnancy loss, or termination related to vertical CMV transmission. This reflects the characteristics of those meeting current clinical criteria for diagnostic CMV testing in the UK and possibly the characteristics of those most motivated to respond to a consultation on screening.

Decisions about screening for cCMV need to consider the criteria for population level screening and its potential impact on the whole screened population. These are discussed in detail in the full UK NSC recommendations^12^ and are outside the scope of this paper. However, the perspectives of respondents to this consultation are crucial for understanding existing gaps in care, the ways that CMV screening decisions are perceived by those living with a diagnosis, and the wider impact of cCMV and health policy on children and families. These respondents’ voices also make an important contribution to the limited literature on lived experiences of children with cCMV and their families, with relevance for related health policies, and wider service development and improvement.

Many families described delays in their child receiving a cCMV diagnosis despite early symptoms, and attributed this to the low awareness of cCMV they had encountered among healthcare professionals, as reported elsewhere.^25^ Under ascertainment of symptomatic cCMV is also apparent in UK hospital and surveillance data,^26,27^ and is likely compounded by the fact that many symptoms of cCMV are non-specific (such as petechiae, jaundice, and small for gestational age).^26^ In this context, families saw screening as a way for diagnosis to be achieved more quickly and for child outcomes to be improved, a view also expressed among parents with lived experience of CMV/cCMV in Canada.^28^ Studies on the psychological impact of rare diseases in infancy and childhood on parents and caregivers highlight the negative impact of “prolonged diagnostic odysseys” that families frequently face, with the period from symptoms to first diagnosis the most psychologically challenging.^29^ In the responses we analysed, the process of seeking a diagnosis was described as distressing for families and burdensome for children, with uncertainty about the causes of symptoms an important source of fears and anxiety.

A key impact of diagnostic delays described by respondents was the missed opportunity for antiviral treatment to be considered for their child, and diagnostic delay was the most common reason for late treatment initiation among children with cCMV in a recent clinical audit.^30^ Where indicated, antivirals would be recommended to commence within the first month of life, although more recent observational evidence has shown possible benefits when initiated up to 3 months of age.^5^ cCMV diagnostic delays beyond 1 or 3 months of age can occur due to lack of recognition of symptoms, but also due to sequential referrals and investigations for symptoms before cCMV diagnostic testing is completed. This is an issue that early cCMV detection pathways in paediatric audiology aim to mitigate, but almost half of paediatric audiology departments in a recent audit in England did not have this pathway available, potentially leading to inequalities in timing of diagnosis and therefore access to treatment.^11^ In addition to missed opportunities for antiviral treatment, respondents identified missed opportunities for early interventions. A cCMV diagnosis is a gateway to appropriate monitoring, including regular audiological follow-up,^5^ given that cCMV can cause delayed-onset, fluctuating and deteriorating hearing loss in early childhood which is particularly frequent among children who are symptomatic at birth.^4^ Early detection and rehabilitation of hearing loss in childhood is crucially important to a wide range of developmental outcomes and quality of life.^31^ Respondents’ beliefs that opportunities had been missed to avert more serious symptoms or impairment led to an enduring sense of injustice, unfairness and having been let down by the health care system.

A further dimension to the emotional impact expressed by parents was the missed opportunities for healthcare professionals to give better support, with links between this and quality of life of the family and child affected by cCMV. Some responses suggested unrealistic expectations (e.g., that newborn screening and treatment of child with cCMV would prevent all cCMV-related sequelae), also indicating unmet need for support and information. A lack of information and specialist knowledge at the time of diagnosis and convoluted health care systems were both found to contribute to psychological distress of parents and caregivers of children with rare diseases in a recent systematic review.^29^ In the UK, families affected by cCMV can access advice and support from CMV Action (https://cmvaction.org.uk/), but delays in diagnosis also delay families accessing this specialist support. There is a research gap around the role of clinical psychology in supporting families affected by cCMV. In a recent feasibility study of a clinical psychology intervention at a tertiary referral centre, parents of children with cCMV showed improved understanding and confidence in managing their child’s needs and gave positive feedback following the intervention, although their wellbeing continued to be variable, possibly linked with ongoing uncertainty about their child’s prognosis.^32^

An important strength of our analyses is the relatively large number of responses available for analysis and their richness. The consultation was open to people throughout the UK, providing the opportunity for some geographical representativeness, although we were unable to confirm this due to the lack of demographic details for respondents. One key limitation is that those experiencing the more severe consequences of cCMV are overrepresented in the sample. A further limitation relates to the use of secondary data; many of the responses were framed in terms of views on screening rather than describing experiences more generally, due to the nature of the consultation. Further to this, we could not follow up with respondents to clarify the meaning of their responses, and some important information was missing (e.g., it was often unclear whether respondents’ comments on screening referred to screening in pregnancy or newborns). The year of birth of affected children referred to in the responses was not collected, however in some cases this was many years previously, and may not reflect contemporary experiences.

In conclusion, although the primary purpose of the UK NSC consultation process was to shape screening policy, we have shown the additional value of responses to this consultation for research. The important gaps in awareness of cCMV and healthcare for affected children highlighted through these analyses have relevance for policy development in related areas (for example, training about cCMV for clinicians). Irrespective of national screening policy decisions, addressing these gaps is essential to improving experiences, access to support and interventions for children with symptomatic cCMV, as well as their families and caregivers. Prospective collection of data for children diagnosed with cCMV through cCMVnet Registry (https://ccmvnet.org/registry/) is an opportunity to explore key metrics such as presence of symptoms at birth and age at diagnosis over time. Responses indicated areas in which parents’ views need to be taken into consideration, in particular the emotional, psychological and quality of life impact of cCMV for children and their families and influence of health care access, an area in which literature is sparse. Findings indicate the need for interventions to address awareness gaps, improve access to information and support, reduce diagnostic delays and to consider the psychological support needs of families and caregivers.

## Data Availability

All data analysed in this manuscript are publicly available through the UK National Screening Committee website (https://view-health-screening-recommendations.service.gov.uk/review/cytomegalovirus-2021-review/download-documents/cover_sheet/).
